# Integrating ECG and echocardiography to identify early-stage transthyretin amyloid cardiomyopathy

**DOI:** 10.1093/ehjimp/qyag058

**Published:** 2026-04-03

**Authors:** Ashwin Venkateshvaran, Per Lindqvist, Björn Pilebro

**Affiliations:** Department of Diagnostics and Intervention, Clinical Physiology, Umeå University, Norrlands Universitetssjukhus, Byggnad 5A, Målpunkt H62, Umeå 901 85, Sweden; Department of Diagnostics and Intervention, Clinical Physiology, Umeå University, Norrlands Universitetssjukhus, Byggnad 5A, Målpunkt H62, Umeå 901 85, Sweden; Department of Diagnostics and Intervention, Clinical Physiology, Umeå University, Norrlands Universitetssjukhus, Byggnad 5A, Målpunkt H62, Umeå 901 85, Sweden; Department of Public Health and Clinical Medicine, Umeå University, Umeå, Sweden

**Keywords:** transthyretin amyloidosis, ATTR-CM, DPD scintigraphy, heart failure, echocardiography, global longitudinal strain, early diagnosis, wall thickness, SaVR, cardiac biomarkers

## Abstract

**Aims:**

Current diagnostic criteria for transthyretin amyloid (ATTR) cardiomyopathy (ATTR-CM) emphasize increased left ventricular (LV) wall thickness, potentially delaying recognition of early myocardial involvement. We evaluated whether combining ECG (S-wave amplitude in lead aVR, SaVR) with echocardiographic relative apical sparing (RELAPS) enhances the detection of ATTR-CM in patients with absent or mild LV hypertrophy.

**Methods and results:**

In this single-centre, retrospective observational study, 39 ATTR patients (72 [64–78] years; 45% female) with interventricular septal thickness ≤13 mm were included. Diagnosis was confirmed by 99mTc–DPD scintigraphy and/or biopsy. Diagnostic performance of RELAPS–SaVR was tested against two comparator groups: patients with mild LV hypertrophy and no evidence of amyloidosis, in addition to age-matched healthy controls with normal cardiovascular status. Peripheral neuropathy was the dominant clinical presentation (66%, *n* = 26), while overt cardiomyopathy presented in a minority (28%) of patients, consistent with early-stage disease. LVEF was preserved in 92%, and restrictive filling was absent. RELAPS ≥1 and SaVR <7 mm was observed in 87% and 85%, respectively. Individually, RELAPS (AUC 0.83) and SaVR (AUC 0.85) distinguished ATTR-CM from LVH. The combined RELAPS–SaVR model improved discrimination (AUC 0.90), with sensitivity 86%, specificity 94%, positive predictive value 76%, negative predictive value 97%, and overall accuracy 89%. Likelihood ratio testing confirmed its incremental value over RELAPS alone (LR = 19.54; *P* < 0.001).

**Conclusion:**

In this single-centre retrospective study, integrating SaVR with RELAPS improved the detection of ATTR-CM in patients with absent or mild LV hypertrophy. These findings support a simple, non-invasive strategy for early detection that merits prospective validation.

## Graphical Abstract

**Graphical Abstract:**
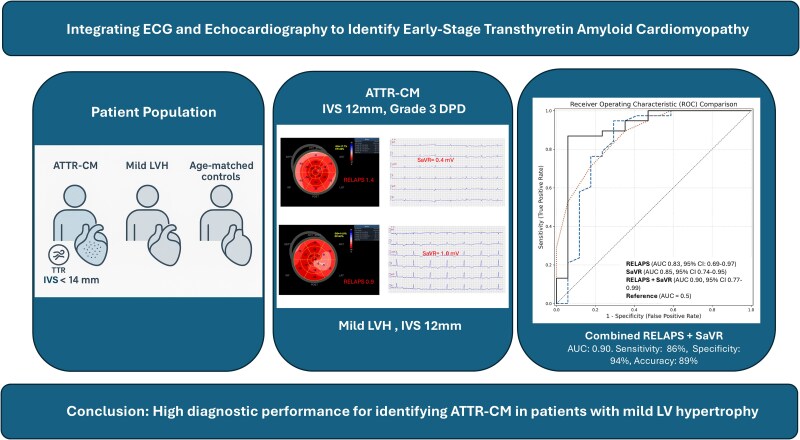

## Introduction

Transthyretin amyloid (ATTR) cardiomyopathy (ATTR-CM) is recognized as an underdiagnosed cause of heart failure in older adults. Despite advances in non-invasive imaging and the emergence of disease-modifying therapies, diagnostic delay remains common, often spanning several years from symptom onset.^1^ Current diagnostic algorithms emphasize left ventricular (LV) wall thickening, with clinically accurate criteria recommending an interventricular septal thickness (IVS) ≥14 mm as a key threshold to raise suspicion for cardiac amyloidosis^2,3^ However, recent echocardiographic consensus statements suggest that a lower LV wall thickness in the absence of alternative causes should also raise suspicion in early or pre-hypertrophic disease^4^ particularly when interpreted alongside the high disease-specificity of DPD-positivity.^5^

Early identification of ATTR-CM is clinically relevant, as current therapies do not induce disease regression but rather slow or halt disease progression.^6–8^ Earlier studies using CMR tissue characterization, including native T1 mapping and extracellular volume (ECV) quantification, have demonstrated that myocardial amyloid infiltration can be detected before overt hypertrophy, highlighting the potential of multimodal approaches for early disease detection.^9^ However, few studies have systematically characterized cardiac structure and function in patients with early-stage ATTR-CM and normal or only mildly increased LV wall thickness, and data on the diagnostic performance of early disease markers remain limited.

The relative apical sparing ratio (RELAPS) is a well-established strain-based echocardiographic screening marker for ATTR-CM.^10^ However, studies suggest that its sensitivity is reduced in early or pre-hypertrophic disease, when the characteristic apical sparing pattern may not yet be fully developed.^11^ This limitation underscores the need for complementary markers to improve detection in patients with absent or mild hypertrophy. Emerging evidence suggests that electrical alterations may precede overt structural remodelling, reflecting early interstitial amyloid deposition before measurable hypertrophy develops.^12^ This creates a clinically relevant diagnostic window in which subtle electrical attenuation and strain abnormalities may provide complementary information.

In this single-centre, proof-of-concept, observational study, we sought to investigate whether combining an electrocardiographic marker (S-wave amplitude in lead aVR, SaVR) with a strain-derived echocardiographic marker (RELAPS) improves diagnostic discrimination for ATTR-CM in patients with mild or absent LVH. We hypothesized that this dual parameter approach would enhance diagnostic accuracy before overt hypertrophy develops.

## Methods

### Patient population

We performed a single-centre, retrospective observational study of patients with ATTR-CM and IVS thickness ≤13 mm at the time of diagnostic work-up between 2006 and 2023 at Umeå University Hospital. Patients with pacemakers, significant arrhythmia, left bundle branch block (LBBB), or significant (>2+) valvular regurgitation were excluded. Diagnosis of ATTR amyloidosis was established either by histologic confirmation on peripheral tissue biopsy using Congo red staining with TTR immunohistochemistry, or non-invasively by 99mTc-DPD scintigraphy after exclusion of AL amyloidosis through blood and urine testing for monoclonal proteins.^5^ Two additional cohorts were included for comparison: (1) patients with mild LVH and (2) age-matched healthy controls. The mild LVH cohort (*n* = 19) included patients with IVS thickness 12–14 mm, preserved LVEF, with no evidence of infiltrative cardiomyopathy or significant valvular disease. The healthy group (*n* = 30) comprised individuals with normal ECG and echocardiographic findings, no history of cardiovascular disease and no use of cardiovascular medicine. These groups served to evaluate diagnostic accuracy and contextualize findings against normal and early structural phenotypes.

### DPD scintigraphy and genotyping

Myocardial tracer uptake was visually graded on planar DPD scintigraphy using the Perugini scale (0–3). Assessment was made by two experienced specialists in nuclear medicine, and there were no disagreements. Transthyretin (TTR) gene sequencing was performed in all patients.

### Echocardiography

Comprehensive transthoracic echocardiography was performed by experienced echocardiographers using Vivid 7, E9, or E95 ultrasound systems (GE Healthcare, Horten, Norway) according to ASE/EACVI recommendations.^13^ Images were exported and analysed offline (EchoPac v204, GE Ultrasound, USA). Standard measurements included LV wall thickness in diastole (LVDD), IVS, and posterior wall thickness (PW) measured in the parasternal long axis view. LV ejection fraction (LVEF) employing the biplane modified Simpson’s method. Relative wall thickness (RWT) was assessed as 2x (PW/LVDD).

### Assessing RELAPS using deformation imaging

Speckle-tracking–derived global longitudinal strain (GLS) was obtained from apical three-, four- and two-chamber views. Strain was semi-automatically calculated by placing three fiduciary points at the apical and basal LV segments, generating a region of interest (ROI) that tracked myocardial motion. Manual adjustments were applied to optimize segmental tracking and to match the ROI width to myocardial thickness. Each view was divided into six segments, and the average GLS was calculated. RELAPS was defined as the ratio of mean apical strain to the mean of basal and midventricular segmental strains.

### Electrocardiography

Standard 12-lead ECGs recorded during the same visit were analysed for rhythm, voltage attenuation, and conduction disturbances. QRS voltage was quantified. S-wave amplitude in lead aVR (SaVR) was measured manually from the ECG tracing at a paper speed of 25 mm/s and calibration of 10 mm/mV. Measurements were performed blinded to echocardiographic and scintigraphic findings and expressed in millivolts (1 mV = 10 mm).

### Biomarkers

Demographics and data regarding cardiac biomarkers were retrieved from medical records. Blood biomarkers were measured within one month of imaging using a Cobas 800 analyser (Roche Diagnostics Scandinavia). Troponin-T was measured using the high-sensitivity STAT assay, and NT-proBNP levels were determined using the STAT assay, both following the manufacturer’s protocols. Estimated glomerular filtration rate (eGFR) was calculated using the CKD-EPI equation.

### Ethical considerations

The study complied with the Declaration of Helsinki and was approved by the local ethics committee (DNR 2016-435-31M with supplementary application DNR 2018-418-32M and 2021-01196). All patients provided written informed consent for participation, including permission to review their medical records in accordance with local regulations.

### Statistical analysis

Continuous variables are reported as medians with interquartile ranges (25th–75th percentiles) and categorical variables as counts and percentages. ATTR-CM subgroup and ATTR-CM-control comparisons were conducted using the Mann–Whitney U test for continuous variables and Fisher’s exact or chi-square tests for categorical variables, as appropriate. A two-sided *P*-value <0.05 was considered statistically significant. Diagnostic performance of echocardiographic parameters and SaVR for identifying ATTR-CM was assessed using receiver operating characteristic (ROC) analysis and area under the curve (AUC). Optimal cut-off values were determined using the maximal Youden’s J statistic. Sensitivity, Specificity, Positive Predictive Value (PPV), Negative Predictive Value (NPV), and Accuracy were assessed. ROC curves were compared using the DeLong test. A combined diagnostic score was formulated using binary logistic regression with the ATTR-CM status as the dependent variable and RELAPS and SaVR as continuous covariates. Given the exploratory proof-of-concept design and limited sample size, internal validation procedures such as bootstrapping or cross-validation were not performed. The multivariable model was intentionally restricted to two predictors to reduce the risk of overfitting. The incremental value of SaVR to RELAPS was assessed by comparing the model fit of the RELAPS-only (Model 1) with the combined RELAPS–SaVR model (Model 2) using the Likelihood ratio test, with 1 degree of freedom corresponding to the addition of SaVR. Goodness-of-fit for each model was assessed using the Nagelkerke pseudo R^2^. Analyses were performed using IBM SPSS version 28 for Windows (IBM Corp., Armonk, NY, USA).

#### Reproducibility analysis

To assess the reproducibility of strain measurements, RELAPS was re-measured in a random subset of 10 examinations. For inter-observer variability, a second independent observer repeated the measurements, blinded to the original results. Reproducibility was assessed using the intraclass correlation coefficient (ICC) based on a two-way random-effects model with absolute agreement. ICC values were interpreted as: poor (<0.5), moderate (0.5–0.75), good (0.75–0.9), and excellent (>0.9). Both Intraobserver reproducibility (ICC 0.99, 95% CI 0.97–0.99) and interobserver variability (ICC 0.98, 95% CI 0.96–0.99) for RELAPS were excellent.

#### Missing data

Missing data were minimal. SaVR values were unavailable in 4 of 88 participants (4.5%). RELAPS and other primary echocardiographic variables were complete. Logistic regression and ROC analyses were performed using complete-case methodology. Biomarker analyses were conducted on available cases.

## Results

### Population characteristics

From a cohort of 213 patients with ATTR-CM, 39 (17 females,72 [64–78] years at diagnosis) had an IVS thickness ≤13 mm and were included in the analysis. Diagnosis was verified by DPD scintigraphy and/or extracardiac biopsy. Genetic testing classified 3 patients (8%) as having wild-type ATTR (ATTRwt) and 36 (92%) as variant ATTR (ATTRv), predominantly Val30Met (*n* = 32), with additional rare variants (Thr60Ala *n* = 1, Val30Leu *n* = 1, Glu54Leu *n* = 1, Glu89Lys *n* = 1, His88Arg *n* = 1). Abdominal fat pad biopsy was positive for transthyretin amyloid in 22 patients, and the rest were diagnosed non-invasively.^5^

The initial presenting symptom was predominantly peripheral neuropathy, accounting for 66% of cases (*n* = 26). Conversely, cardiac symptoms or abnormal echocardiographic findings were the reason for diagnostic work-up in a small subset (*n* = 11, 28%), consistent with early-stage cardiac manifestation. Presentations of gastrointestinal and ocular involvement were also seen (*n* = 1, 2% each).

Patient Characteristics, echocardiography, and electrocardiography findings in the ATTR-CM cohort, stratified by IVS thickness, are presented in *Table 1*. In the total cohort, 92% (*n* = 36) had preserved LVEF (≥ 50%). None demonstrated restrictive filling (E/A > 2). RELAPS ≥ 1 was observed in 87% (*n* = 34). GLS was reduced (<−16%) in 36% (*n* = 14), and mitral e’ velocity < 5 cm/sec in 25% (*n* = 10). Low SaVR (<7 mm) was seen in 85% (*n* = 33) of the cohort. When stratified into two groups based on the median IVS thickness, ATTR-CM patients with lower wall thickness had lower troponin, NT-proBNP, and higher mitral e’ velocities when compared with those with higher wall thickness (*Table 1*). No differences were observed when LVEF, RELAPS, or SaVR were compared between IVS subgroups (*P* > 0.05 for all comparisons).

**Table 1:** Clinical characteristics, electrocardiography, and echocardiography data of the patient cohort stratified by median wall thickness

	All ATTR-CM ≤13 mm (*n* = 39)	ATTR-CM ≤12 mm (*n* = 19, 49%)	ATTR-CM >12 mm (*n* = 20, 51%)	*P*-value
Age, years	72 [64–78]	70 [63–79]	74 [64–78]	0.49
Female, (%)	18 (45)	11 (55)	7 (35)	0.15
HR, bpm	74 [68–86]	74 [66–84]	80 [70–85]	0.92
Weight, kg	66 [59–79]	61 [54–73]	70 [62–90]	0.09
Height, cm	170 [164–176]	168 [162–175]	171[166–178]	0.27
SBP, mmHg	130 [115–145]	130 [114–148]	130 [115–144]	0.85
DBP, mmHg	80 [74–84]	78 [75–80]	80 [70–85]	0.53
**Laboratory**				
Troponin, ng/L	16 [9–29]	10 [6–18]	20 [14–39]	0.01
NT-proBNP, ng/L	220 [113–732]	150 [92–365]	506 [142–1158]	0.02
eGFR, mL/min/1.73	84 [73–92]	87 [74–92]	81 [68– 90]	0.33
**ECG**				
SaVR, mV	0.50 [0.40–0.70]	0.60 [0.40–0.70]	0.50 [0.30–0.60]	0.19
**Echocardiography**				
IVS thickness, mm	13 [12–13]	12 [11–12]	13 [13–13]	<0.001
PW thickness, mm	9 [8–11]	9 [7–11]	8.5 [8–11]	0.81
LVDD, mm	45 [42–50]	44 [41–48]	47 [43–51]	0.15
RWT, mm	0.40 [0.34–0.46]	0.43 [0.35–0.46]	0.38 [0.32–0.46]	0.36
LV EF, %	55 [55–60]	55 [55–61]	55 [55–60]	0.96
Stroke volume (ml)	75 [67–89]	71 [67–87]	79 [66–91]	0.35
Mitral E/A ratio	0.90 [0.7–1.1]	0.95 [0.72–1.1]	0.90 [0.77–1.0]	0.51
Mitral e’ velocity (cm/s)	6 [5–7]	7 [6–9]	6 [4–7]	0.04
LV GLS, -%	17 [15–19]	18 [15–19]	16 [14–18]	0.22
RELAPS	1.33 [1.14–1.56]	1.30[1.14–1.56]	1.34[1.12–1.66]	0.56
RWT/SaVR	0.75 [0.51–1.08]	0.75 [0.58–0.93]	0.76 [0.50–1.26]	0.55

ATTR-CM = transthyretin cardiac amyloidosis; DBP = diastolic blood pressure; EF = ejection fraction; eGFR = estimated glomerular filtration rate; GLS = global longitudinal strain; HR = heart rate; IQR = interquartile range; IVS = interventricular septum; LBBB = left bundle branch block; LV = left ventricle; LVDD = left ventricular diastolic diameter; NT-proBNP = N-terminal pro–B-type natriuretic peptide; PW = posterior wall; RELAPS = relative apical sparing; RWT = relative wall thickness; SaVR = S-wave amplitude in lead aVR; SBP = systolic blood pressure. *P*-value represents differences between ATTR-CM subgroups.

### Comparison with control groups

When compared with age-matched healthy controls, ATTR-CM patients displayed higher heart rates, lower SaVR, higher RELAPS, and RWT (*P* < 0.05 for all comparisons). When compared with the mild LVH group (IVS = 13 [12–14] mm), ATTR-CM patients displayed lower LVEF, SaVR, and higher RELAPS (*P* < 0.05 for all comparisons) (*Table 2*).

**Table 2:** Clinical characteristics, electrocardiography and echocardiography data of ATTR-CM, age-matched controls and LVH

	All ATTR-CM ≤13 mm (*n* = 39)	Age-matched controls (*n* = 30)	LVH (*n* = 19)	*P*-value ATTR-CM vs. controls	*P*-value ATTR-CM vs. LVH
Age, years	72 [64–78]	67 [67–72]	65 [56–80]	0.13	0.15
Female, (%)	18 (45)	15 (50)	9 (47)	0.20	0.35
HR, bpm	74 [68–86]	64 [60–70]	70 [64–77]	<0.001	0.07
Weight, kg	66 [59–79]	75 [64–83]	76 [70–85]	0.17	0.04
Height, cm	170 [164–176]	173 [163–177]	172 [164–179]	0.90	0.45
SBP, mmHg	130 [115–145]	140 [120–146]	148 [122–176]	0.26	0.07
DBP, mmHg	80 [74–84]	80 [70–82]	76 [68–88]	0.73	0.84
**ECG**					
SaVR, mV	0.50 [0.40–0.70]	0.80 [0.70–1.0]	0.90 [0.67–1.32]	<0.001	<0.001
**Echocardiography**					
IVS thickness, mm	13 [12–13]	10 [9–11]	13 [12–14]	<0.001	0.09
PW thickness, mm	9 [8–11]	8.5 [7–10]	9 [8–10]	0.22	0.92
LVDD, mm	45 [42–50]	50 [48–53]	48 [43–52]	<0.001	0.13
RWT, mm	0.40 [0.34–0.46]	0.35 [0.29–0.40]	0.37 [0.35–0.41]	0.009	0.34
LV EF, %	55 [55–60]	63 [59–66]	68 [55–77]	<0.001	0.01
Stroke volume (ml)	75 [67–89]	79 [69–86]	86 [70–90]	0.99	0.19
Mitral e’ velocity (cm/s)	6 [5–7]	8 [6– 9]	6 [6–10]	0.02	0.18
LV GLS, −%	17 [15–19]	18 [16–19]	19 [15–20]	0.14	0.07
RELAPS	1.33 [1.14–1.56]	0.60 [0.50–0.65]	0.94 [0.63–1.1]	<0.001	<0.001

ATTR-CM = transthyretin cardiac amyloidosis; DBP = diastolic blood pressure; EF = ejection fraction; eGFR = estimated glomerular filtration rate; GLS = global longitudinal strain; HR = heart rate; IQR = interquartile range; IVS = interventricular septum; LBBB = left bundle branch block; LV = left ventricle; LVDD = left ventricular diastolic diameter; NT-proBNP = N-terminal pro–B-type natriuretic peptide; PW = posterior wall; RELAPS = relative apical sparing; RWT = relative wall thickness; SaVR = S-wave amplitude in lead aVR; SBP = systolic blood pressure. *P*-value represents differences between ATTR-CM and controls, and ATTR-CM and LVH respectively.

### Diagnostic performance

Diagnostic performance was assessed by including ATTR-CM and those with LVH as a comparator group. Healthy controls were excluded from the primary analysis to minimize the risk of overfitting, as their inclusion could artificially inflate discrimination. Sensitivity, Specificity, PPV, NPV, and Accuracy for each diagnostic variable at the cut-off determined by Youden’s J Statistic are summarized in *Table 3*. Analyses including healthy controls are also presented in Supplementary data online, *Table S1*.

**Table 3:** Diagnostic performance of echocardiographic variables and SaVR for identifying ATTR-CM: sensitivity, specificity, and accuracy determined by optimal ROC-defined thresholds (Youden's J statistic)

Variable	Cut-off	AUC	CI	*P*-value	Youden index	Sens (%)	Spec (%)	PPV (%)	NPV (%)	Acc (%)
SaVR, mV	≤ 0.70	0.85	0.74–0.95	<0.001	0.50	89	61	82	72	80
RELAPS	≥ 1.0	0.83	0.69–0.97	<0.001	0.59	87	72	87	72	82
EF, %	≤ 55	0.68	0.69–0.86	0.04	0.26	57	68	78	43	61
GLS, -%	≤ 17	0.65	0.48–0.81	0.07	0.21	68	53	75	44	63
RWT	≥ 0.43	0.58	0.43–0.72	0.27	0.23	40	85	84	40	54

AUC = area under the curve; CI = confidence interval; SaVR = S-wave in lead aVR; RELAPS = relative apical sparing ratio; EF = left ventricular ejection fraction; GLS = global longitudinal strain; RWT = relative wall thickness; NPV = negative predictive value; PPV = positive predictive value.

Both RELAPS (AUC 0.83; 95% CI 0.69–0.97) and SaVR (AUC 0.85; 95% CI 0.74–0.95) displayed a strong ability to differentiate ATTR-CM from LVH patients **(***Table 3*). At an optimal threshold of 1.0, RELAPS displayed a sensitivity of 87%, specificity of 72%, and overall accuracy of 82%. For SaVR, an optimal cut-off of 0.7 yielded a sensitivity of 89%, specificity of 61%, and overall accuracy of 80%. In logistic regression models, RELAPS and SaVR were included as continuous covariates. Formal assessment of model assumptions confirmed linearity of the logit (Box-Tidwell test, *P* > 0.05 for all covariates) and absence of collinearity between predictors (VIFs < 2), supporting the validity of the model estimates. Binary logistic regression revealed a strong inverse association between SaVR and the odds of ATTR-CM (Beta = −6.29, SE = 2.1; *P* = 0.003). RELAPS had a positive association (Beta = 1.69, SE = 1.1), although it did not reach statistical significance in the combined model (*P* = 0.13). The combined logistic regression model describing the probability of ATTR-CM was defined as logit (*P*) = −3.149–6.29 (SaVR) + 1.69 (RELAPS).

### Incremental diagnostic value of SaVR to RELAPS

**Figure 1:**
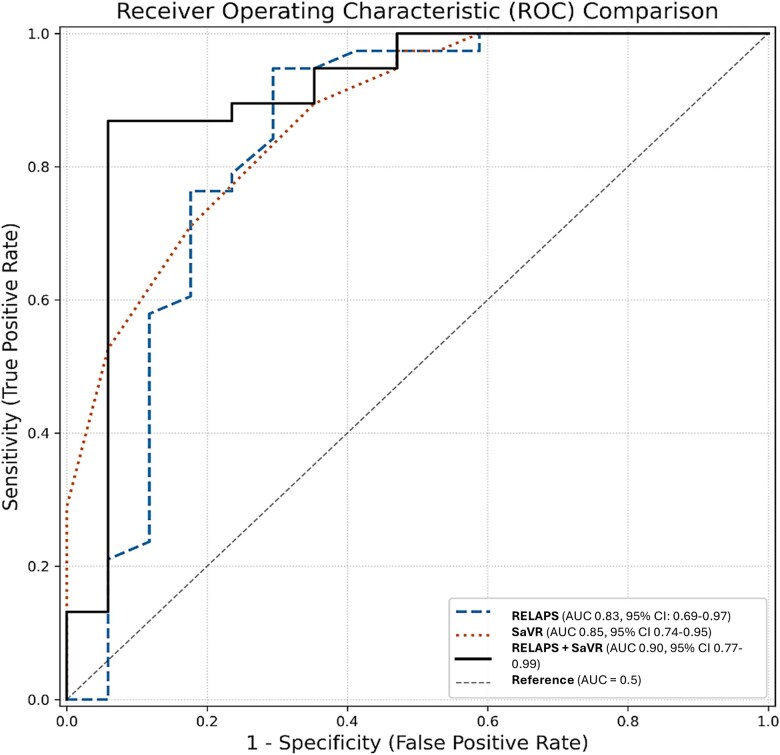
Receiver operating characteristics displaying the superior performance of the combined RELAPS–SaVR model in ATTR-CM with absent or mildly increased wall.

The diagnostic performance of the resulting combined RELAPS–SaVR Score showed significant improvement over the individual scores, achieving an AUC of 0.90; 95% CI 0.77–0.99, sensitivity of 86%, specificity of 94%, PPV of 76%, NPV of 97%, and overall accuracy of 89% (*Figure 1*). The superiority of RELAPS–SaVR when compared with RELAPS was further reaffirmed by the Likelihood Ratio Test = 19.54, df = 1; *P* < 0.001. The goodness-of-fit model increased substantially from 0.23 (RELAPS) to 0.58 (RELAPS–SaVR). The combined RELAPS–SaVR model displayed superior diagnostic performance even when healthy controls were added as comparators (see Supplementary data online, *Table S1*).

## Discussion

In this single-centre, retrospective, proof-of-concept observational study, we demonstrate that combining SaVR with RELAPS enhances discrimination of ATTR-CM among patients with absent or mildly increased wall thickness. In this early structural phenotype characterized by preserved systolic function and relatively low biomarker burden, the integrated model achieved high specificity and negative predictive value. These findings support the concept that electrical and mechanical alterations may precede overt morphologic remodelling and that their integration may facilitate earlier diagnostic recognition. Although validation in larger multicentre cohorts is required, our results provide hypothesis-generating evidence for a pragmatic and scalable approach using routinely available investigations.

### Diagnostic accuracy of RELAPS in ATTR-CM

While multiple studies have established the value of RELAPS as a diagnostic marker in ATTR-CM with markedly increased wall thickness,^10,11^ its sensitivity in early or pre-hypertrophic disease remains debated. CMR-based markers such as native T1 and ECV have demonstrated the ability to detect interstitial amyloid expansion before overt wall thickening, highlighting that early disease may be identifiable through tissue-level changes even when conventional structural parameters remain normal.^9^ The characteristic pattern of apical sparing typically emerges when basal and midventricular segments are disproportionately infiltrated, a stage that may not yet be present in patients with near-normal wall thickness. Previous studies have shown inconsistent results regarding the diagnostic yield of RELAPS in mild hypertrophy, with some reporting limited sensitivity when IVS <14 mm.^11,14,15^ This limitation, compounded with observations that early ATTR-CM often escapes recognition due to preserved LVEF and absence of overt hypertrophy^16,17^ underscores the challenges of detecting early-stage disease. Our data suggest that coupling SaVR to RELAPS may help overcome these limitations.

### Electrical-mechanical integration for ATTR-CM diagnosis

Our group has previously shown that incorporating SaVR with relative wall thickness confers strong diagnostic accuracy to detect ATTR-CM in patients with IVS more than 14 mm.^18,19^ During the pre-hypertrophic stage, electrical remodelling may precede mechanical dysfunction, driven by amyloid infiltration and gap-junction disarray that impair depolarization propagation. Studies evaluating voltage-to-mass ratios and other ECG indices indicate that electrical changes often precede structural remodelling, consistent with the early reduction in SaVR observed in our cohort.^20^ This reduction observed in 85% of our cohort supports this notion and reinforces prior findings that lead aVR may capture diffuse myocardial involvement earlier than traditional voltage indices such as Sokolow–Lyon.^18^ Importantly, these patients were not considered to have less cardiomyopathy only based on septal thickness but also due to normal RWT (<0.5), LVEF (>50%), stroke volume (>66 mL), and mitral E/A ratio (< 1) despite abnormal DPD uptake.

The superior performance of the combined model is likely rooted in its ability to capture the electrical–mechanical discordance characteristic of early amyloid deposition. (1) The strong complementary relationship between RELAPS and SaVR provides incremental diagnostic value beyond either parameter alone. The combined RELAPS–SaVR model achieved an AUC of 0.90, outperforming conventional echocardiographic indices, which typically range from 0.70–0.85 for distinguishing ATTR-CM from other LVH aetiologies.^21^ This underscores its potential utility for risk enrichment, although prospective validation is required before clinical rule-out strategies can be recommended. Importantly, the RELAPS–SaVR approach also parallels recent efforts to integrate multimodal metrics and AI-assisted strategies to better delineate early amyloid cardiomyopathy, combining electrical and mechanical signals.^22,23^

### Genotype-specific implications of RELAPS–SaVR

The predominance of the Val30Met variant in our cohort may have influenced these findings. In ATTR Val30Met amyloidosis, neurological manifestations often precede overt LV hypertrophy, with subtle echocardiographic findings and relatively preserved systolic function at diagnosis.^24^ Two pathways for amyloidogenesis have been suggested in this population, one in which amyloid is formed from full-length TTR and one in which amyloid formation involves fragmentation of TTR.^25^ The former leads to a mainly neurological disease, whereas the latter causes cardiac involvement indistinguishable from that seen in other ATTRv populations and ATTRwt amyloidosis, in addition to polyneuropathy.^26^ The mixed phenotype ATTRv amyloidosis allows for the study of early stages of ATTR-CM, but whether the results can be directly translated to ATTRwt amyloidosis warrants further validation in larger, genotype-diverse populations.

### Clinical and translational implications

From a clinical standpoint, combining SaVR and RELAPS offers a simple, accessible, and cost-effective approach to refine early detection of ATTR-CM. Both parameters can be obtained from routine ECG and standard echocardiography, making them well suited for integration into existing or AI-based diagnostic workflows for heart failure, infiltrative cardiomyopathy, or unexplained LV thickening.^3,4^ Further, incorporating these indices into automated reporting systems could facilitate prompt further confirmatory work-up.^27,28^ By identifying patients before structural remodelling and systolic decline occurs, this approach may extend the therapeutic window for treatment initiation or optimization with disease-modifying therapies and improve long-term outcomes.

#### Limitations

This study has several limitations. First, it is a retrospective, single-centre analysis with a modest sample size, which limits statistical power and external validity. Because model derivation and performance assessment were conducted in the same dataset, diagnostic performance may be subject to optimism bias and requires validation in independent external cohorts. Our findings should therefore be interpreted as hypothesis-generating and require validation in larger, prospective, multicentre cohorts. Second, to ensure reliable ECG voltage and strain measurements, we excluded patients with pacing, advanced conduction disease, or significant arrhythmias. While methodologically necessary, these exclusions may introduce selection bias and limit applicability to real-world ATTR-CM populations, where conduction abnormalities and rhythm disturbances are common. Third, the predominance of the Val30Met variant in our cohort constrains generalizability. Genotype-specific differences in disease expression may influence early electrical and mechanical remodelling patterns, and the performance of the RELAPS–SaVR approach in ATTRwt and other variant populations warrants further investigation. Fourth, the LVH comparator group was relatively small and not comprehensively phenotyped for all alternative causes of hypertrophy. Although major structural heart diseases were excluded based on clinical evaluation and imaging review, residual confounding from unrecognized aetiologies cannot be entirely excluded. Fifth, a formal assessment of inter-observer variability for DPD scintigraphy was not performed. While all scans were evaluated by experienced nuclear medicine physicians using standardized Perugini grading, potential variability between observers cannot be excluded. Finally, while RELAPS demonstrated utility in our models, its specificity may be reduced in the presence of comorbid conditions such as chronic kidney disease, which affects the generalizability of the diagnostic model.

## Conclusion

Integrating SaVR with RELAPS enhances diagnostic accuracy for early identification of ATTR-CM among patients with absent or mild LV hypertrophy. Prospective validation in multicentre cohorts is warranted to support integration into multimodal diagnostic workflows for early diagnosis and timely therapeutic intervention.

## Lead author biography


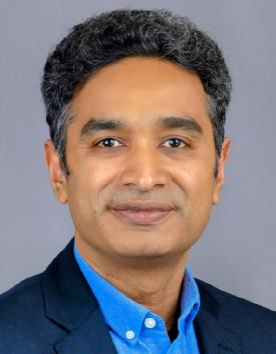


Ashwin Venkateshvaran, PhD, FASE, FESC, is Associate Professor of Clinical Physiology and a Senior Staff Scientist at Umeå University, Sweden. His research focuses on advanced cardiac imaging – including echocardiography, cardiac MRI, PET, and invasive haemodynamics––to improve early diagnosis, disease phenotyping, and risk stratification in heart failure and infiltrative heart disease.

## Supplementary Material

qyag058_Supplementary_Data

## Data Availability

Data underlying this article can be shared upon reasonable request to the corresponding author.
