# Supplementary material for: Integrating ECG and echocardiography to identify early-stage transthyretin amyloid cardiomyopathy
Source: Eur Heart J Imaging Methods Pract. 2026 Apr 3;4(1):qyag058. doi: 10.1093/ehjimp/qyag058 (PMC13089482; doi:10.1093/ehjimp/qyag058)
Supplement: qyag058_Supplementary_Data [file qyag058_supplementary_data.docx]

**SUPPLEMENTAL TABLES**

**SUPPLEMENTAL TABLE 1.** Diagnostic Performance of echocardiographic variables and SaVR for Identifying ATTR-CM with LVH + healthy controls as comparators: Sensitivity, Specificity, and Accuracy Determined by Optimal ROC-defined Thresholds (Youden's J Statistic)

| **Variable** | **Cut-off** | **AUC** | **CI** | **P-value** | **Sens (%)** | **Spec (%)** | **PPV (%)** | **NPV (%)** | **Acc**  **(%)** |
| --- | --- | --- | --- | --- | --- | --- | --- | --- | --- |
| SaVR, mV | ≤ 0.65 | 0.86 | 0.78 – 0.94 | <0.001 | 72 | 84 | 78 | 79 | 78 |
| RELAPS | ≥ 1.0 | 0.93 | 0.88 – 0.99 | <0.001 | 87 | 89 | 87 | 89 | 88 |
| EF, % | ≤ 55 | 0.74 | 0.64 – 0.85 | <0.001 | 59 | 83 | 74 | 72 | 73 |
| GLS, -% | ≤ 17 | 0.62 | 0.50 – 0.74 | 0.04 | 48 | 61 | 50 | 60 | 55 |
| RWT | ≥ 0.43 | 0.64 | 0.52 – 0.76 | 0.01 | 41 | 87 | 72 | 65 | 67 |
| RELAPS + SaVR | ≤ 0.29 | 0.96 | 0.91 – 1.00 | <0.001 | 86 | 98 | 97 | 90 | 93 |

**Abbreviations:** AUC = area under the curve; CI = confidence interval; SaVR = S-wave in lead aVR; RELAPS = relative apical sparing ratio; EF = left ventricular ejection fraction; GLS = global longitudinal strain; RWT = relative wall thickness; NPV = negative predictive value; PPV = positive predictive value.
